# Convention of Western Medical Schools

**Published:** 1849-01

**Authors:** 


### CONVENTION OF WESTERN MEDICAL SCHOOLS.

We earnestly hope this project will not meet with the fate that has heretofore attended similar propositions. The medical schools are contemplating lengthened sessions; but have they yet devised any expedient for keeping the students to the end of the present term? Is it not now notorious that the classes begin to break up soon after they are formed, and that many students, from mere impatience of confinement, are wending their way to their distant homes soon after the Christmas holidays? Here is an evil confessed, palpable, enormous, for which certainly it would not be difficult to devise a remedy. A convention of the western schools, there is reason to believe, would lead to the adoption of measures which would correct it. Our brethren beyond the mountains do not appear to be troubled in this way, but there can be no doubt that they would co-operate with us in the enforcement of any regulation which would advance the general cause of medical education in the country.

